# Anti-viral state segregates two molecular phenotypes of pancreatic adenocarcinoma: potential relevance for adenoviral gene therapy

**DOI:** 10.1186/1479-5876-8-10

**Published:** 2010-01-29

**Authors:** Vladia Monsurrò, Stefania Beghelli, Richard Wang, Stefano Barbi, Silvia Coin, Giovanni Di Pasquale, Samantha Bersani, Monica Castellucci, Claudio Sorio, Stefano Eleuteri, Andrea Worschech, Jay A Chiorini, Paolo Pederzoli, Harvey Alter, Francesco M Marincola, Aldo Scarpa

**Affiliations:** 1 Department of Pathology, University of Verona Medical School, Verona, Italy; 2 ARC-NET Center for Applied Research on Cancer, The Verona Hospital Concern and The University of Verona, Verona, Italy; 3 Infectious Disease and Immunogenetics Section (IDIS), Department of Transfusion Medicine, and Center for Human Immunology (CHI), National Institutes of Health, Bethesda, MD, USA; 4 Gene Therapy and Therapeutics Branch, National Institute of Dental and Craniofacial Research, National Institutes of Health, Bethesda, Maryland, USA; 5 Department of Surgery, University of Verona Medical School, Verona, Italy

## Abstract

**Background:**

Pancreatic ductal adenocarcinoma (PDAC) remains a leading cause of cancer mortality for which novel gene therapy approaches relying on tumor-tropic adenoviruses are being tested.

**Methods:**

We obtained the global transcriptional profiling of primary PDAC using RNA from eight xenografted primary PDAC, three primary PDAC bulk tissues, three chronic pancreatitis and three normal pancreatic tissues. The Affymetrix GeneChip HG-U133A was used. The results of the expression profiles were validated applying immunohistochemical and western blot analysis on a set of 34 primary PDAC and 10 established PDAC cell lines. Permissivity to viral vectors used for gene therapy, Adenovirus 5 and Adeno-Associated Viruses 5 and 6, was assessed on PDAC cell lines.

**Results:**

The analysis of the expression profiles allowed the identification of two clearly distinguishable phenotypes according to the expression of interferon-stimulated genes. The two phenotypes could be readily recognized by immunohistochemical detection of the Myxovirus-resistance A protein, whose expression reflects the activation of interferon dependent pathways. The two molecular phenotypes discovered in primary carcinomas were also observed among established pancreatic adenocarcinoma cell lines, suggesting that these phenotypes are an intrinsic characteristic of cancer cells independent of their interaction with the host's microenvironment. The two pancreatic cancer phenotypes are characterized by different permissivity to viral vectors used for gene therapy, as cell lines expressing interferon stimulated genes resisted to Adenovirus 5 mediated lysis in vitro. Similar results were observed when cells were transduced with Adeno-Associated Viruses 5 and 6.

**Conclusion:**

Our study identified two molecular phenotypes of pancreatic cancer, characterized by a differential expression of interferon-stimulated genes and easily recognized by the expression of the Myxovirus-resistance A protein. We suggest that the detection of these two phenotypes might help the selection of patients enrolled in virally-mediated gene therapy trials.

## Background

The incidence and mortality of pancreatic ductal adenocarcinoma (PDAC) almost coincide and novel therapeutic approaches are needed for this deadly disease. Gene therapy aimed at the delivery of gene functions capable of enhancing cancer cell immunogenicity [1] or inducing oncolysis is a promising approach [2-6].

Viral vectors well suit the purpose of gene therapy and adenoviruses are commonly used gene-delivery vectors due to the efficiency of their in vivo gene transfer [7]. Since 1993, about 300 clinical trials based on adenoviral vectors have been performed [8]. However, a significant limitation to their utilization is the host's immune response [9].

Physiologically, a viral infection stimulates the synthesis of interferons (IFNs) that are then secreted to activate the innate immune response of uninfected neighboring cells preventing the viral spread. This endogenous immune response is induced by the recognition of viral components by Toll-like receptor agonists [10,11] and follows a two-step process, consisting in the induction of type I IFNs followed by the transcriptional activation of hundreds of IFN-stimulated genes (ISGs) [12]. In turn, the activation of ISGs promotes the rapid expression of proteins with direct anti-viral function such as the Myxovirus-resistance-A (MxA) protein that protects infected as well as non-infected bystander cells [13] against a wide variety of viruses including adenovirus [14].

Various cancers including melanoma, breast, head and neck, prostate, lung and glioma display transcriptional profiles that suggest the existence of two subgroups of cancer cells distinguishable according to a characteristic IFN and inflammatory chemokines expression pattern [15-20]. Interestingly, Weichselbaum et al. [20] recently reported that IFN-related DNA damage resistance signatures occur in common human cancers and can predict responsiveness of breast cancer to chemotherapy and radiation therapy based on the expression pattern of ISGs.

In this study, we identified by transcriptional profiling two ISG-defined phenotypes of pancreatic cancer that are readily recognized by immunohistochemistry according to the expression of MxA as a marker of IFN activity. The two phenotypes display diverse permissivity to adenoviral replication in vitro suggesting the practical implication that these signatures could facilitate the identification of patients likely to respond/resist viral vector-delivered gene therapy.

## Methods

### Pancreatic cancer samples

Thirty-four primary PDAC and 10 established PDAC cell lines from the Biobank of the Department of Pathology, University of Verona were used following approval by the institutional Ethics Committee. The 34 samples comprised 23 primary bulk PDAC tissues and 11 primary PDACs that were cancer-cell enriched by xenografting PDAC tissues in athymic nu/nu mice [21]. The 10 human PDAC cell lines included Panc1, MiaPaCa-2, HPAF-I, CFPAC1, Ger, PSN1, Panc2, Paca3, Paca44 and PT45 [22].

### Microarray analysis

RNA from 8 xeno-grafted primary PDAC, 3 primary PDAC bulk tissues, 3 chronic pancreatitis and 3 normal pancreatic tissues was hybridized to a GeneChip HG-U133A containing 22,283 probe sets (21,430 genes, Affymetrix, Sacramento, CA). RNA quality and concentration were assessed using Agilent 2100 Bioanalyzer (Agilent Technologies, Palo Alto, CA). First- and second-strand cDNA were synthesized from 12.5 μg of total RNA according to manufacturer's instructions (Affymetrix). After in vitro transcription, labeling and fragmentation, probes were hybridized to the GeneChips that were then washed in a GeneChip Fluidics Station 400 (Affymetrix); results were visualized with a Gene Array scanner using Affymetrix software. Array data were normalized and summarized using the RMA method [23]http://bioconductor.org/packages/2.0/bioc/src/contrib/affy_1.14.0.tar.gz. Cluster analysis was based on cluster and Treeview software (Eisen's laboratory, Berkeley, CA). Functional interpretations were based on Gene Ontology and Ingenuity Pathways Analysis software http://www.ingenuity.com.

### Western Blot analysis

Western blot analysis using MxA (sc-50509, Santa Cruz Biotechnology Delaware, CA) and β-actin (sc-47778, Santa Cruz Biotechnology) antibodies was performed on 11 primary xenografted PDAC, 4 primary PDAC bulk tissues, 1 normal pancreatic tissue and 10 PDAC cell lines. Antibodies against MxA and β-actin were used at a dilution of 1:1000 and 1:2000, respectively. As positive control for MxA expression, peripheral blood mononuclear cells from healthy donors were incubated overnight with IFN-alpha at a final concentration of 100 IU/ml.

### Immunohistochemical analysis

A tissue microarray (TMA) containing 23 primary PDACs, 11 xenografts, and 3 normal pancreas was stained with MxA antibody (sc-50509, Santa Cruz Biotechnology). The TMA was constructed using 1 mm cylinders from selected areas of formalin-fixed paraffin-embedded tissues using a tissue micro-arrayer from Beecher Instruments (Sun Prairie, WI). Four tissue cores were arrayed for each sample. Three μm sections were de-paraffinized, boiled for 30 min at 98°C in 10 mM citrate buffer pH 6, treated with 3% hydrogen peroxide 10 min and then with Protein Blocking Agent (Novocastra Laboratories, Newcastle, UK) for 10 min. MxA antibody was applied diluted 1:1000 for 60 min at room temperature. Sections were washed and treated with NovoLink Polymer Detection System according to manufacturer's instructions (Novocastra).

### Cell line culture, infection, and transfection with BAAV vector

Ad5-CMV-GFP and Ad5-CMV-null were purchased from Applied Viromics (Fremont, CA). AAV5 and AAV6 were from Dr J.A. Chiorini. Ad5-Luc was a gift of Zheng, Changyu (NIH/NIDCR, Bethesda, MD). Cells were cultured in RPMI 10% FBS in 6-well plates at 2 × 10^5 ^until 70% confluence, washed twice with cold phosphate buffered saline (PBS) and infected overnight at 37°C in 5% CO2 with Ad5-CMV-GFP or Ad5-Null as at 13 pfu/cell (10×) or 136 pfu/cell (100×). Media was replaced after 24 hours and cells expressing GFP were observed after 2 days under a fluorescence microscope (Zeiss Axiovert 200 M - Software: Openlab). On day 2, cells were trypsinized, washed with 2 ml FACS Buffer (PBS plus 2,5% FBS), at 1,200 rpm for 5 minutes at +4°C and fixed with 4% paraformaldehyde. Cyto-fluorimetric analysis was performed using FACS Canto cyto-fluorimeter and the FACS Diva software (Becton Dickinson, San Jose, CA) while the supernatant after lysis was collected for testing viral load by real time qPCR. AAV infection was performed in Costar black 96 well plates with clear flat bottom (Corning, NY). Luciferase assay was performed using the Bright-Glo lysis buffer/substrate (Promega, Madison, WI).

293T human kidney cells were maintained in Dulbecco's modified Eagle's medium: recombinant AAVs expressing EGFP or LUC were produced using a four-plasmid procedure as previously described [24]. The AAV particle titers were in the range of 10^12 ^DNAse resistant particles (DRP) × ml. Adenovirus type 5 wt from crude lysate titer and Ad DNA replication was determined by qPCR using the following primers: Ad type 5 forward primer 5'-AACCGAAGGCTGCATTCACT, reverse primer 5'-ACCGCACAGGGTCTTAATAGAG. Following denaturation at 96°C for 10 min, cycling conditions were 96°C for 15s, 60°C for 1 min for 40 cycles. The viral DNA in each sample was quantified by comparing the fluorescence profiles with a set of Ad DNA standards (449B plasmid).

Plasmids for constructing pISRE-SEAP and pIFN-beta-SEAP, and pMetLuc-Control were obtained from Clontech. Secreted alkaline phosphatase (SEAP) and secreted luciferase from Metridia were selected for reporter assays. The human IFN-beta promoter -281- to +20 sequence (Genbank # EF064725) was synthesized by GenScript and confirmed by DNA sequencing. pIFN-beta-SEAP was constructed by sub-cloning human IFN-beta promoter into pTAL-SEAP. Plasmid pISRE-SEAP and pNFkB-SEAP were similarly constructed into the pISRE-Luc. SEAP reporters were under the control of IFN-stimulated response element (ISRE) and IFN-betapromoter in pISRE-SEAP and pIFN-beta-SEAP, respectively. Cells transfected with pMetLuc-control plasmid expressed and secreted luciferase constitutively in the tissue culture media under the control of CMV IE promoter and were used as internal control for normalization of the transfection efficiency. Phospha-Light™ SEAP Reporter Gene Assay System was obtained from Applied Biosystems (Foster City, CA). Ready-To-Glow Secreted Luciferase Reporter System for Metridia secreted luciferase (Met-Luc) was obtained from Clontech (Mountain View, CA).

Cells were seeded at 2.5 to 3 × 10^5^/well into 6-well plates, grown overnight, then washed with 2 ml Opti-MEM I reduced serum medium (Invitrogen, Carlsbad, CA) and fed with 1 ml of the same medium. Transfections were conducted using Lipofectamine 2000 transfection reagent (Invitrogen) with 4 μl of Lipofectamine. Reporter plasmids (0.5 μg pIFN-beta-SEAP, pISRE-SEAP, or negative control vector pGeneClip) and internal control vectors (10 ng pMetLuc-control) were diluted in 250 μl of Opti-MEM I, then added into the lipofectamine mixture and incubated for an additional 20 min. The lipofectamine/DNA mixture was added to each well, incubated at 37°C for 4 h and aspirated. Treated wells were fed with 3 ml complete RPMI medium without antibiotics, and incubated for 20-24 h. Culture supernatants were collected to assay the activities of SEAP and Met-Luc by chemi-luminescence. SEAP activity was normalized to Met-Luciferase activity. Data were expressed as mean relative SEAP unit. The fold induction of promoter activity was calculated by dividing the normalized SEAP activity from pIFN-beta-SEAP or pISRE-SEAP transfected cells with that of control plasmid transfected cells (relative activity).

### RNA Interference Assay

Small interfering RNAs (siRNA) for interferon regulatory factor IRF-3, IRF-7, virus-induced signaling adapter (VISA), and the non-targeting control (NC) siRNA were obtained from Ambion (Austin, TX). NF-kB p65 siRNA was obtained from Cell Signaling Technology (Danvers, MA). For detailed information about the sequences please refer to additional File 1. Transfection of siRNAs was carried out using Lipofectamine 2000 (Invitrogen) at a final concentration of the siRNA mixture at 50 nM. Cells transfected with siRNAs were further incubated for 36-48 hrs and then reporter gene plasmids were introduced into cells and the culture supernatant were collected for chemi-luminescence assays.

## Results

### IFN-related signatures suggest the existence of two molecular phenotypes of PDAC

Eight xenografted primary PDACs, three primary PDAC bulk tissues, three chronic pancreatitis and three normal pancreatic tissues were hybridized to a 21,430 gene GeneChip HG-U133A Affymetrix array.

**Figure 1:**
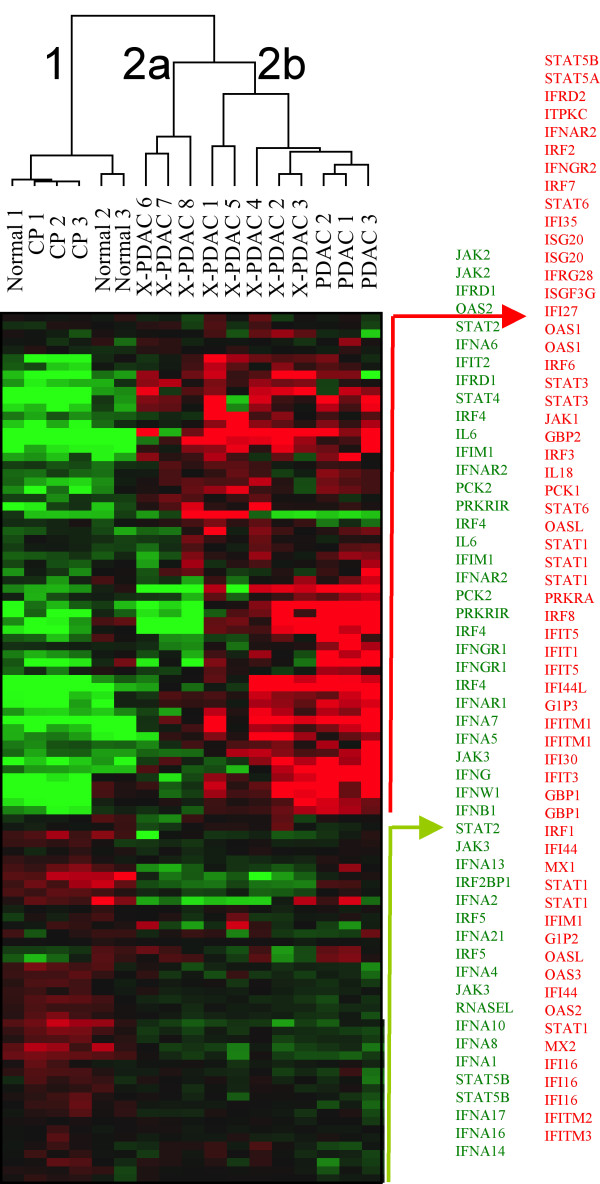
**Interferon related genes expression profile**. Supervised cluster expression analysis of 76 selected interferon related genes, represented by 112 probesets, in 8 xenografted primary pancreatic adenocarcinomas (X-PDAC), 3 pancreatic adenocarcinoma bulk tissues (PDAC), 3 chronic pancreatitis (CP) and 3 normal pancreas (Normal). The analysis distinguished a cluster comprising the 11 adenocarcinoma samples (cluster 2) from the normal and pancreatitis samples that clustered together (cluster 1). Among the cancer samples there were two phenotypes, 2a and 2b, the former being closer to the cluster of normal and pancreatitis. The list of probesets corresponding to up regulated genes in group 2b is listed in red while those corresponding to down regulated genes are in green.

Class comparison identified a module enriched of ISGs among the genes differentially expressed by PDACs compared to normal tissues or pancreatitis. We, therefore, selected from the complete data set 76 genes, represented by 112 probesets, associated with IFN signaling according to Gene Ontology such as IFNs, IFN receptors, IFN regulatory factors (IRFs), IFN stimulated genes (ISGs), IFN induced proteins (IIPs), IFN associated signaling pathway molecules, such as JAK and STAT and IFN associated proteins, such as IL18 and OAS molecules (additional file 2). Hierarchical clustering using this gene set identified two main clusters (Figure 1, additional file 3), the first including normal pancreas and chronic pancreatitis (cluster 1), the second including all the PDACs (cluster 2). Moreover, two subgroups could be identified within cluster 2, the first including three xenografts (cluster 2a) and the other (cluster 2b) including the five remaining xenografts and the three PDAC bulk tissues.

**Cluster 2b displayed a profile diametrically opposite to that of normal pancreas or chronic pancreatitis **and was characterized by upregulation of ISG and IIP genes, while all IFN (including IFN-alpha4,5,7,17, IFN-beta1, IFN-omega1) and several IFN receptor genes (including IFN-alpha, beta and omega receptor 1, IFNalphabeta and omega receptor 2) were down regulated. Display of the IFN canonical pathways by Ingenuity Pathway Analysis showed that IFN-related genes were activated predominantly down-stream of IFN receptor/IFN interactions (additional file 3). As the activation of ISGs typically follows a viral infection, we considered these tumors as bearing an "anti-viral state".

**Figure 2:**
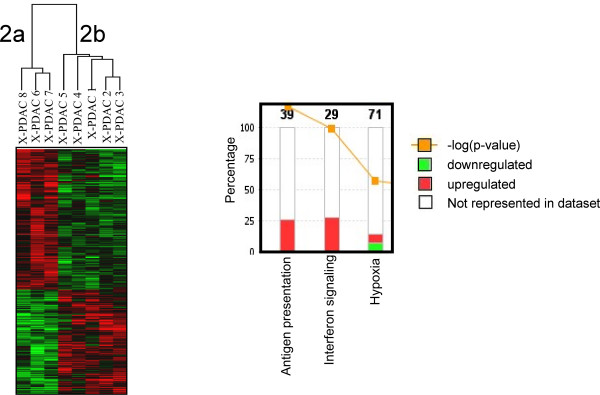
**Genes differentially expressed between clusters 2a and 2b xenografts**. Left panel, cluster analysis of 1,203 differentially expressed genes between the clusters 2a and 2b of Figure 1 (red indicates up-regulation while green down-regulation). Right panel, canonical pathway analysis of the 1,203 genes using the Ingenuity Pathway Analysis software. The 3 most significantly modulated pathways are indicated; the stacked bars represent the proportion of differentially expressed genes over the total number of genes involved in the specific pathway (number on top of the bars).

To characterize the difference between the two cancer phenotypes, we examined the genes differentially expressed between cluster 2a and 2b and found that a set of 935 genes were differentially expressed at a broad cut-off of significance (*Student's *T test p_2 _< 0.05) (Figure 2, additional file 4). This low threshold of significance was selected to include all genes of potential relevance for pathways analysis [25,26]. To verify the relevance of the gene selection in spite of the low significance threshold a permutation test [27,28] was performed following NCI criteria [29] demonstrating that this assortment reflected a true biological difference rather than resulting stochastically from the large number of tests. Ingenuity Pathway Analysis confirmed predominant up regulation of genes associated with IFN signaling (but not IFN or IFN receptor) as well as human leukocyte antigen (HLA) class I and class II genes (Figure 2) and genes related to antigen processing. Interestingly, the hypoxia pathway was also differentially affected (Figure 2). Among genes associated (i.e. IL18, OAS genes) or directly involved in IFN signaling (JAK/STAT), STAT1 and OAS1, OAS2, OAS3 and MxA best distinguished the two phenotypes.

### MxA expression discriminates the two ISG-related molecular phenotypes of PDAC

**Figure 3:**
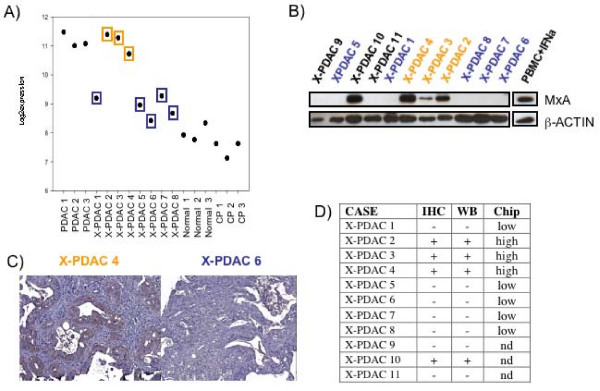
**MxA protein expression in xenografted primary pancreatic adenocarcinomas**. A) MxA expression level in microarray data analysis expressed as log2 ratio; orange and blue colors represent higher and lower expression transcript, respectively. B) Western Blot analysis of MxA in 11 xenografted primary pancreatic adenocarcinomas (X-PDAC). C) Example of MxA immuno positive (X-PDAC 4) and MxA immuno negative (X-PDAC 6) samples. D) Correlation of MxA immunohistochemistry, Western Blot and microarray data.

Among the ISGs differentially expressed between the two PDACs phenotypes, MxA was selected as marker for the "anti-viral phenotype" since this protein is directly associated with anti-viral properties [30]. Individual display of MxA transcription is reported in Figure 3A, protein expression by Western Blot in Figure 3B and by immunohistochemistry in Figure 3C. MxA expression by immunohistochemical and Western blot were concordant with transcriptional analysis showing that four of 11 xenografts (36%) displayed an anti-viral phenotype (Figure 3D).

**Figure 4:**
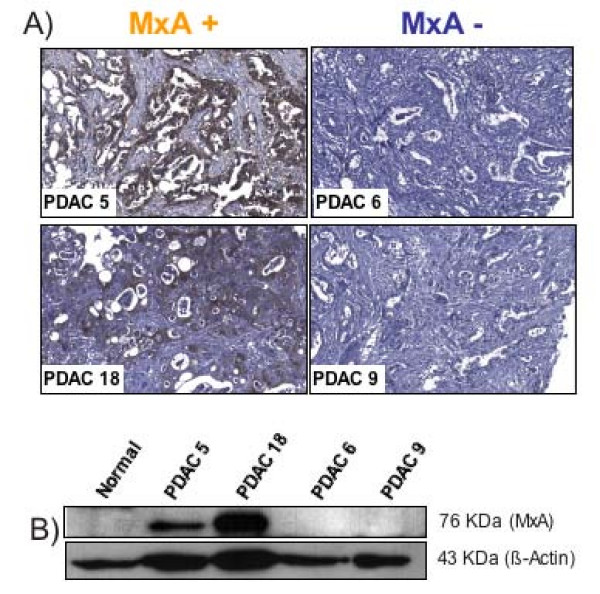
**MxA protein expression in primary pancreatic adenocarcinoma tissues**. Immunohistochemical (A) and Western blot (B) analysis of MxA in four primary pancreatic adenocarcinomas (PDAC).

The existence of two diverse molecular phenotypes of PDAC based on the expression of MxA was confirmed in an independent set of 23 primary PDACs by immunohistochemistry. Ten (43%) PDACs stained positively for MxA (Figure 4A); three had over 80% of cancer cells expressing MxA while seven had a positivity ranging from 25% to 60%. Western Blot of four of these primary PDACs confirmed the findings with two MxA-positive and two MxA negative samples (Figure 4B).

### Adenoviral infection of PDAC cell lines

**Figure 5:**
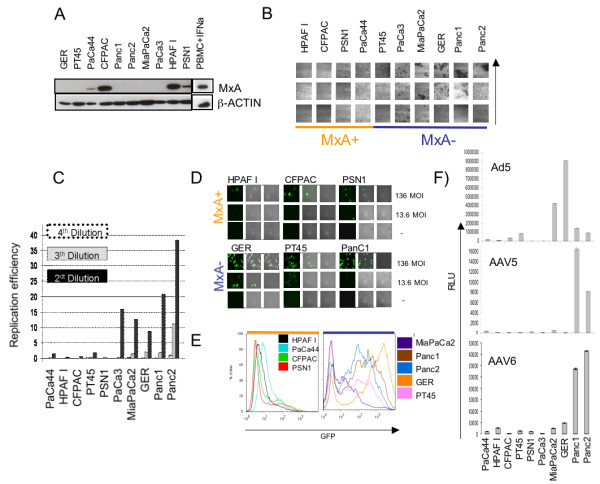
**Endogenous MxA expression in PDAC cell lines and resistance to viral infection**. A) MxA expression in PDAC cell lines by Western Blot analysis. B) Citotopathic effect of Adenovirus wt on MxA+ (orange) versus MxA- (blu) PDAC cell lines. The vertical arrow indicates increased viral concentration, from 10^6^, 10^7^, 10^8 ^DNA particles of Ad5. C) Number of viral particles measured by real time PCR after Adeno5 wt infection in MxA+ and MxA- PDAC cell lines (Ad5 DNA replication efficiency). Normalised to the Ad5 DNA amount present in Panc2 at 4^th ^dilution considered as 1 Correlation of MxA expression with Adeno5 infection efficiency. MxA positive (HPAFI, CFPAC, PSN1, top) and MxA negative (GER, PT45, Panc1, bottom) cells were infected with 1.36 pfu/cell, 13.6 pfu/cell and 136 pfu/cell of Ad5-CMV-GFP vector. D) FACS analysis profile of different PDAC cell lines after 2 days of Adeno5-CMV-GFP infection (13.6 pfu/cell). E) Luminescence analysis for the permissivity of MxA+ and MxA- to the adeno associated infection, data are shown as relative luciferase units (RLU).

To assess the functional relevance of the anti-viral state, we screened 10 PDAC cell lines for MxA expression. Western Blot analysis discriminated cancer cell lines into MxA positive (PaCa44, HPAFI, CFPAC, PSN1) or MxA negative (Ger, PT45, Panc1, Panc2, MiaPaCa2, PaCa3) (Figure 5A). These lines were tested in an in vitro assay for permissivity to Adenovirus replication or transduction using a wild type or recombinant virus frequently used as oncolytic and gene therapy vectors for experimental cancer therapies. Cell lines that did not express MxA were more prone to the cytopathic effects and more permissive to viral replication than those expressing MxA (Figure 5B and 5C). PDAC transduction by serial dilution of Ad-GFP resulted also in higher expression of GFP in lines not expressing MxA (Ger, PT45, Panc1, Panc2, MiaPaCa2) (Figure 5D and 5E).

### Adeno-Associated viral infection of PDAC cell lines

To assess whether MxA expression influences cancer cell permissivity to the infection by viruses other then adenovirus, we tested the transduction properties of the Adeno Associated Virus (AAV) types 5 and 6 on 8 representative PDAC cell lines (Figure 5F). In spite of intrinsic trophic differences between AAV type 5 and 6, the relative transduction properties of the two viruses is quite similar. Also in this case, cell lines expressing MxA were much less prone to transduction than MxA negative cells.

### Antiviral status is partially depending on IRF7

**Figure 6:**
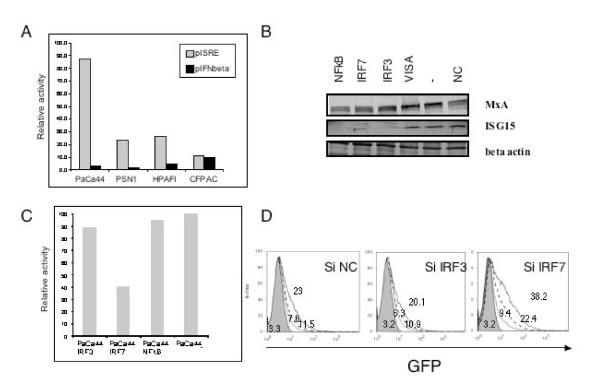
**Silencing and infection with Adeno5 of a MxA positive cell line: PaCa44**. A) Activation of ISRE promoter (gray bars) and IFNbeta promoter (black bars) in MxA+ cell lines. The Y axes express the production of the reporter gene normalized by the same cell line carrying a plasmide with non-targeting control (NC). Please add n of experiments and error bars The data were normalized using a pMet luc plasmid control. B) ISG15 expression by Western Blot after 24 hours of silencing for NFkB, IRF7, IRF3, VISA, untreated, non-targeting control (NC), respectively. C) Decreased level of ISRE regulated reporter gene expression in PaCa44 cell line after silencing with IRF3, IRF7, NFkB or non-targeting control (NC). The data were normalized using a pMet luc plasmid control. D) FACS analysis profile of GFP expression in PaCa44 cell line infected with Adeno5 CMV-GFP virus after silencing IRF3 and IRF7. Cells were infected by using 136 pfu/cell: solid black line, 68 pfu/cell: dashed black line, 27.2 pfu/cell: dotted black line of Adeno5-CMV-GFP vector and 136 pfu/cell Adeno5-CMV-Null vector: solid grey. Numbers represent the MFI.

To assess the permanent activation of the ISGs, we transfected the MxA positive PDAC cell lines with two plasmids, one with an alkaline phosphatase regulated by the ISRE promoter, and a second with an alkaline phosphatase regulated by the IFN-beta promoter. As shown in Figure 6A all four MxA-expressing cell lines demonstrated spontaneous activation of the ISRE promoter independently of external stimulus while no constitutive activation for the IFN-beta promoter was seen.

To confirm that the endogenous activation of ISG was responsible for the reduced permissivity to viral infection, we silenced transcription factors known to be associated with viral resistance. We focused on one MxA positive cell line, the PaCa44, and used the ISG15 gene, directly dependent on ISRE promoter, as a marker of downstream silencing (Figure 6B). Silencing NFkB, IRF3 and IRF7 but not VISA (Figure 6B) decreased expression of ISG15 probably due to the decreased activity of ISRE promoter as also monitored by the decreased production of reporter gene in transfected cells at least for IRF7 (Figure 6C). Though NFkB, IRF7 and IRF3 silencing decreased ISG15 expression, only IRF7 decreased the level of the reporter gene expression by more than 50% (Figure 6C) and partially reverted the resistance to infection with Ad5GFP (Figure 6D).

## Discussion

It has been reported that melanoma metastases display a heterogeneous phenotype in vivo that could be segregated according to the coordinate expression of an inflammatory signature including cytokines, chemokines and angiogenic factors [16,31]. The expression of these genes followed a modular behavior and was coordinated among them resulting in two cutaneous melanoma metastases phenotypes. Modular "operon-like" gene expression has been recognized to be a relatively common feature in several immune pathologies [20,32] and may offer a bottom up view of complex diseases and their interaction with the host. The original observation described for metastatic melanoma could not separate the identified modular patterns between those related to the host's response to cancer cells and those primarily due to potential taxonomic differences between two molecular subsets of cutaneous melanoma [33].

The present study confirms this phenomenon, and in addition suggests that 1) the two phenotypes ("inflammatory" vs "quiescent") are not limited to cutaneous melanoma but are also present in pancreatic adenocarcinoma, suggesting that it could be possibly a widespread phenomenon among cancers; 2) the activation of ISGs is due to two independent taxonomies of cancer cells and not to the host's reaction to the cancer as it is was observed in xenografts growing in immune deficient animals and in *in vitro *cultured cell lines; 3) the two phenotypes reflect a true "anti-viral" state capable of inhibiting replication of at least two families of viruses (adeno viruses and adeno associated viruses); 4) the two cancer taxonomies described here may bear relevant biological characteristics that might affect treatment of cancer with viral vectors or with immunotherapy.

It remains to be elucidated why these two phenotypes exist. One possibility is that the cancer cells bearing the "anti-viral" state are chronically infected with a latent virus that could induce endogenous activation of innate cellular immune responses. Alternatively, it might represent an endogenous activation of anti-viral pathways associated with the mutagenic process. This phenomenon has been clearly described for Epstein-Barr virus or papilloma virus related cancers and could apply to other viruses as well [34,35]. However, two observations mitigate against this interpretation. First, no genes encoding for any known type I IFNs were observed to be up-regulated in association with the "anti-viral state" or the down-stream activation of ISGs; although type one IFN expression is not an absolute requirement for ISG activation during cytomegalovirus infection [36], this IFN-independent activation of ISGs remains to be demonstrated in other viral models in which IFN production at mRNA and protein levels are believed to be crucial [30,37]. Second, in a preliminary analysis, we compared a number of cancer cell lines bearing either phenotype by hybridizing their mRNA to a commercially available pathogen chip containing probes for all known viruses (Agilent Technology) and we could not identify any viral sequence in the cell lines (Worschech A et al., unpublished observation).

Thus, the "anti-viral state" is a characteristic molecular phenotype of a subset of pancreatic cancers that may be the result of a specific mutational profile of cancer cells which is difficult to be understood at this time [38]. Epigenetic level control, such as methylation, may represent an additional mechanism since a strict correlation exists between demethylation and enhancements in STAT-1 phosphorylation followed by an increase in ISG expression [39]. From the gene ontology analysis it was interesting to observe the participation of hypoxia pathways in cancer cells with the "anti-viral" state as this can clearly affect tumor biology and responsiveness to chemotherapy [40] and likely immunotherapy of immune responsive cancers such as renal cell carcinoma [41] and melanoma [42].

We could also speculate that the constitutive activation of antigen presentation pathways might be significant in modulating T cells responses and be responsible for their heterogeneity in various cancers; this may explain the immunogenicity of some melanomas compared with other melanomas [43] and may become a tool to stratify cancer patients to be treated with T cell-directed vaccines. Whether cancer cells with an active "anti-viral" state bear an enhancement in the presentation of endogenous proteins needs to be evaluated in future studies.

The existence of cancer cells with "anti-viral" capacity has potential relevance to viral gene therapy approaches. Adenoviruses and Adeno-Associated viruses are used to deliver genes to tumor cells with the goal of modifying the phenotype, as for example, by introducing suicide genes [44,45]. Particularly in the case of incurable solid tumors such as pancreatic adenocarcinoma, trials have been initiated with third generation adenoviral vectors [46,47]. The present study suggests that gene delivery by adenoviral vectors might be hampered in some patients; this information can be important in the selection of patients undergoing virally-related gene therapy and could provide important insights into the interpretation of clinical results.

Brunicardi's group [48] demonstrated that gene therapy using Adenovirus subtype 5 mediates rat insulin promoter directed thymidine kinase (A-5-RIP-TK)/ganciclovir (GCV) gene therapy resulting in significantly enhanced cytotoxicity to both Panc1 and MiaPaCa2 pancreatic cancer cells *in vitro *[49]. An *in vivo *study from the same group showed that systemically administered A-5-RIP-TK/GCV is an effective treatment for pancreatic cancer [50]. These studies are based on a rat PDAC model in which the pancreatic tumors were derived from Panc1 and MiaPaca2 cell lines. In this model they found a very tight correlation among A-5-RIP-TK/GCV cytotoxicity to malignant cells, adenoviral dose and length of GCV treatment [48]. Interestingly, all the experiments were performed on cell lines that were negative for the MxA expression. These findings are in full accordance with our theory of a possible effect of interferon associated gene up regulation and its relationship to gene therapy outcome.

If these findings are confirmed in humans, positivity for MxA at diagnosis might become important exclusion criteria and might consequently increase the efficacy of viral-mediated gene therapy for those who test MxA negative.

The observation that both Adenovirus and Adeno Associated viruses were similarly affected by the anti-viral state suggests that this phenomenon is at least partially independent of viral idiosyncrasies related to specific receptors or other restricted properties of each individual virus but rather is a general phenomenon that can apply to several oncolytic delivery systems. Of course, work needs to be done to assess the relevance of this phenotype in other viral systems.

The existence of either phenotype in xenografted primary cancers and *in vitro *models provides evidence that the antiviral state phenotype is stable. Since most of those genes are expressed only during viral infection in non cancer patients, this observation makes some of the product of those inducible genes, for example ones that codify for membrane proteins, new markers and new possible therapeutic target.

## Conclusions

Our findings stress the *in vivo *occurrence in human adenocarcinoma of two distinct phenotypes based on expression of ISGs. Those phenotypes might be important for the resistance to possible introduction of genes using viral vectors or for the resistance to oncolytic gene therapy. We believe that this finding can be of crucial interest for the field of cancer vaccines and gene therapy by giving important pre-screening tools that could aid in the selection of patients most likely to benefit. Alternatively, understanding this resistance mechanism could provide a new target for anti-cancer drug development.

## List of Abbreviations

AAV: adeno-associated virus; CP: chronic pancreatitis; IFN: interferon; IIP: interferon induced protein; IPA: ingenuity pathway analysis; IRF: interferon regulatory factor; ISG: interferon stimulated genes; MxA: myxovirus-resistance A; PDAC: pancreatic ductal adenocarcinoma; TMA: tissue microarray; X-PDAC: xenografted primary pancreatic ductal adenocarcinomas

## Competing interests

The authors declare that they have no competing interests.

## Authors' contributions

VM outlined the study, Ad5GFP infection and sketched the manuscript. SBeg characterized samples and organized validation studies on human samples. SBar designed the microarray experiment and performed data normalization. RW designed the plasmid for transfections and carried out silencing experiments. MC, SC and SE performed western blot analysis, part of silencing experiments and helped sketch the manuscript. JAC coordinated and GDP performed the AAV infections and Ad5 oncolytic virus. SBer performed cryostat enrichment of primary cancers, RNA preparation and immunohistochemical assays. CS created xenografted primary cancers. AW performed the IPA analysis. PP coordinated the recruitment of patients and surgical samples. HA critically revised the experimental plans and the manuscript. FMM conceived and designed the study and validation experiments in vitro. AS contributed to study conception, designed the expression profiling and validation experiments on tissue samples, and finalized the manuscript. All authors read and approved the final manuscript.

## Supplementary Material

Additional file 1 **Sets of siRNA duplexes used for silencing experiments**. List of siRNAs to silence IFR3, IFR7 and VISA. Click here for file

Additional file 2 **Expression levels of genes associated with IFN signaling**. List of 112 probesets representing 76 genes associated with IFN signaling classified according to their predominant expression in either neoplastic or non neoplastic tissues. Click here for file

Additional file 3 **Cellular localization and expression status of the genes listed in **Figure 1**that participate to the canonical interferon pathways (elaboration with Ingenuity Pathway Analysis)**. In red, genes up regulated in cluster 2 vs cluster 1; in green, genes down regulated in cluster 2 vs cluster 1. Click here for file

Additional file 4 **Differentially expressed genes in MxA-positive xenografts vs Mxa-negative xenografts**. List of 935 differentially expressed genes. Click here for file
