# Supplementary material for: Anti-viral state segregates two molecular phenotypes of pancreatic adenocarcinoma: potential relevance for adenoviral gene therapy
Source: J Transl Med. 2010 Jan 29;8:10. doi: 10.1186/1479-5876-8-10 (PMC2845551; doi:10.1186/1479-5876-8-10)
Supplement: Additional file 1 — Sets of siRNA duplexes used for silencing experiments. List of siRNAs to silence IFR3, IFR7 and VISA. [file 1479-5876-8-10-S1.DOC]

**Additional File 1: Sets of siRNA duplexes used for silencing experiments** (sense strand sequence):

**IRF3**

siRNA ID number 115222 (5’-CCCACAUAAAAUCUACGAGUU-3’)

siRNA ID number 106518 (5’-GGAAGGAGGVGUGUUUGACUU-3’)

**IRF7**

siRNA ID number 115481 (5’-CCCAGUCUAAUGAGAACUCUU-3’)

siRNA ID number 41888 (5’-GUGCAAGGUGUACUGGGAGUU-3’)

**VISA**

siRNA ID number s33179 (5’-CUGUUGCAUUGGUCCCUGAUU-3’)

siRNA ID number s33180 (5’-GGGUUCUUCUGAGAUUGAAUU-3’)

siRNA ID number s33178 (5’-CCAAAGUGCCUACCACCUUUU-3’)
