# Supplementary figures and images for: Anti-viral state segregates two molecular phenotypes of pancreatic adenocarcinoma: potential relevance for adenoviral gene therapy
Source: J Transl Med. 2010 Jan 29;8:10. doi: 10.1186/1479-5876-8-10 (PMC2845551; doi:10.1186/1479-5876-8-10)

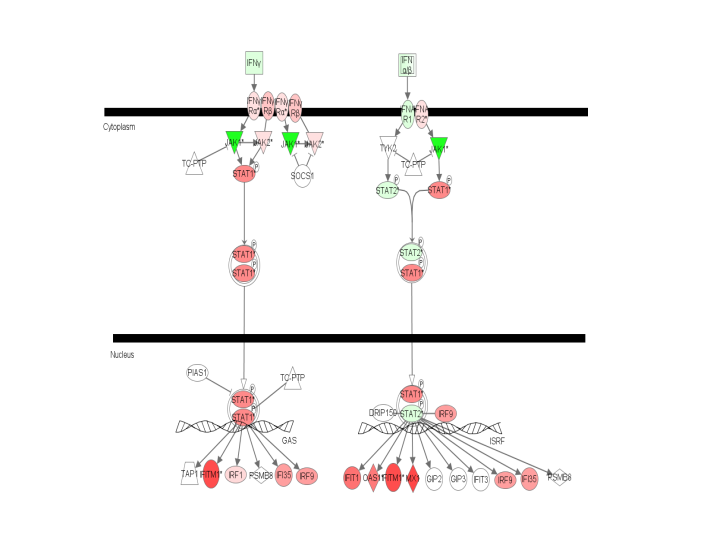

Supplement: Additional file 3 — Cellular localization and expression status of the genes listed in Figure 1that participate to the canonical interferon pathways (elaboration with Ingenuity Pathway Analysis). In red, genes up regulated in cluster 2 vs cluster 1; in green, genes down regulated in cluster 2 vs cluster 1. [file 1479-5876-8-10-S3.PNG]
